# Pennsylvania Hospital

**Published:** 1839-01-12

**Authors:** 


### CLINICAL LECTURE.

### PENNSYLVANIA HOSPITAL.

### DELIRIUM TREMENS.

*Wednesday, January 2d.*—Dr. Coates remarked:

Gentlemen: I propose to occupy your attention this morning with a few remarks on mania-a-potu, or delirium tremens. I shall be unable to introduce any patients labouring under this disease, as the consequent excitement would be highly prejudicial to them. This inability, however, I the less regret, as you have had frequent opportunities of witnessing the disease in the wards, and familiarizing yourselves with it. This is a disease of much interest to the practitioner, from its frequent occurrence in this country. It is also one in which it is peculiarly necessary that the mind of the practitioner should be well made up and decided, as his doubt, fluctuation, and uncertainty, materially increase the mortality. Fifteen years ago it was called exclusively mania-a-potu; at present, the profession are divided between it and the term delirium tremens, which is, in fact, a far more appropriate appellation. The disease is not of the nature of mania, but of delirium; hence the impropriety of the first name. From the chief symptom of the disease being the seeing of phantoms, it might, perhaps, with more propriety, be called *delirium phantasmagoricum.* When a man has been for a number of years addicted to the constant use of ardent spirits, we find the functions of his organs materially impeded, and frequently suspended. After death, the stomach and small intestines show traces of inflammation, and the liver is often extensively diseased. The brain and its membranes are implicated. These are the effects of habitual drinking, but do not constitute delirium tremens. When a man, accustomed to the habitual stimulus of a large quantity of liquor, suddenly leaves off, he experiences, for a day or two, a variety of uneasy sensations, and craves stimulants. If no remedy be used, in the course of a few days, he becomes unable to sleep at night, is generally affected with tremors, and imagines a variety of men, animals, and other objects, to surround him. It is not uncommon for such a patient to appear pretty well during the day,—and the physician is, from this cause, particularly liable to be taken by surprise—it being difficult to imagine that the individual who appears perfectly rational and quiet at the time of conversing with him, had been in a state of high derangement the preceding night, and, perhaps, attempted to throw himself out of the window. This state of things is sometimes repeated for several successive nights, before it becomes established during the day.

Tremors are not essential to the disease, and I have seen it frequently without them,—but the imagining of phantoms is really characteristic. Hence, the impropriety, as has been alleged by able writers, of deriving the name from tremors, and my reason for calling it *delirium with phantoms*, it being, perhaps, unnecessary to make a new Greek English word for the purpose.

One of the most peculiar features of this affection, is the insusceptibility of patients labouring under it to bodily pain. A patient with a fractured leg, will, unless prevented, rise from his bed, and stump about the room in his splints, without feeling the laceration of his flesh by the broken points of bone. If the delirium supervenes on a pleurisy or peritonitis, the pain of these will not be experienced until the nervous system is tranquillized and returns to its natural condition.

It appears evident to me, that the brain is in a state of excessive and continued action,—an action not only continuing all night, while others sleep, and taking cognizance of all real objects presented to it, but coining unreal images. Looking on this disease, as I do, as consisting essentially in a morbid activity of the brain, which does not permit the necessary repose to that organ, the object to be accomplished in its cure, is to reduce this unnatural cerebral excitement.

If this be adequately effected, sleep will ensue, precisely as it is matter of common experience to see it occur in any other individual who has passed several nights without it. This I consider as an *index* of the patient's approach nearer to a natural condition. This does not appear to me to have anything hypothetical about it, but to be the expression of a notorious fact. Is it not matter of common sense that a man in health, if deprived of sleep for several nights in succession, if left undisturbed, will sleep immediately? I do not deny that sleep may also be a *cause* of tranquillity to the mind; but it is of most importance as an index.

This morbid excitement is certainly not identical with inflammation, and is in practice most conveniently reduced by means of opium. In 1818, while an interne of this hospital, having the responsibility of two important cases thrown on me, I was induced to push the opium treatment to an extent considerably beyond what was at that time usual with us, and with the gratification, apparently, of saving two lives which were rapidly approaching a fatal termination. The quantity of opium given, was, in one case, forty-five grains, in seven hours; and, in the other, 400 drops of laudanum. Extreme care is necessary, and the physician should watch the consequences every time he gives five or six grains. The largest quantity I have ever given, so as to produce its whole effect at one time, is fifty-two grains. I am informed that physicians of high qualifications have carried it as far as seventy-two, and one hundred and twenty grains, but I have not done so. I do not mean to express any condemnation of the practice, but simply to state the fact that it has not happened to me to do so. I have been taxed with relying upon opium exclusively. This has never been the case, as could be easily proved. I feel that some apology is due for taking up your time with what is personal to myself; but something may be allowed to a claim of justice, and there is an additional reason for denying it, as from your attendance here, gentlemen, an error on this point might mislead you. Unquestionably, various other remedies will contribute to the desired end. Some practitioners, as I understand then, employ ardent spirits as a part of the means to *cure* the disease. To this I object; apprehending that ardent spirits cannot produce the effect of tranquillizing the mind, without producing a tendency to sleep; and further, conceiving them badly adapted to this task, as producing sleep by such means, appears to me identical with making the patients dead drunk. I do not think, unless there be no better, or *equally good* means in our power, that we have even a moral right to do this.

Understand me, then, to say, that I do not approve of administering ardent spirits, with the view of curing the disease or producing sleep; but where patients are weak, and require them from that cause, I would give brandy and wine, just on the same principles as I would in typhus fever, where I knew inflammations were present, but also knew, from experience, that these substances were useful, and served to sustain the powers of life. From my own experience, I should say, that, including all cases of delirium with phantoms, from the first attack to the fourteenth or twentieth, about twenty-five per cent. of them required ardent sprits, for a period exceeding an hour or two, and the remaining seventy-five either required less than this, or were better without it. To this must be added the bad moral effect of allowing patients and their friends to say that the physician found it necessary.

Blood-letting has been employed by some physicians, but the general experience of the profession is against it. I have never seen it used to any extent without a fatal termination. It was formerly thought here that blood-letting tended to bring on the disease. I do not think so; but am abundantly convinced, for myself, that it does not, in the slightest degree, ameliorate the disease; that death rarely takes place in any other way than by exhaustion of the strength; and blood-letting, by weakening the powers of life, accelerates the end. Add to this, that the patient is, in general, debilitated by previous excesses.

I observe a dependence on depletion recommended by a German and an Italian physician of high character,* based, as far as I have seen, on morbid anatomy. The morbid appearances, which are undoubtedly those of a gastro-entero-arachnitis, or more correctly, as I apprehend, and following Georget, a gastro-enteritis with a superficial encephalitis, would certainly lead to this conclusion any one who should study this disease in a dissecting room, and not in a hospital. I observe, too, that Professor Warren, of Boston, states, in a recent paper, that he found the mortality less in a number of cases which were bled, than in others.

I have the most profound respect for the character of Dr. Warren, and should feel extremely grieved to be found wanting in this regard; but am bound by the moral tie, to tell what I really believe to be correct. Dr. Warren does not give the details of the cases, or inform us by what symptoms they were characterized as delirium tremens, or on what grounds they were selected for bleeding. The patients must have been different from ours, either in the circumstances of the disease, in constitution, climate, local habits, or in some other important particulars; for sure I am that this practice, if persevered in, is with us a short and certain road to death. It is a matter of solemn and unhappy experience that the mortality is greatly increased by bleeding. I will not undertake to assert that bleeding is never necessary, but am sure it is very rarely so.

Leeches and cups are often serviceable. It is sometimes the better plan, in the application of cups, to cut but a few of them, and let the others be dry. Blisters to the back of the neck are often of much use. In the six cases we have been treating, the opium was never given beyond the amount of eighteen grains in the twenty-four hours; and I believe that beyond twenty-eight grains it rarely requires to be carried. Camphor and assafœtida are found valuable adjuvants. The objection to the assafœtida, is its liability to vomit. Morphia is preferred by some to opium. With me it has not been so successful; but this may have occurred from personal reasons, from my greater familiarity with opium, and therefore greater confidence in the use of it. In this hospital, in cases of mania, with violent excitement, no practice has been more successful in producing a rapid diminution of the excitement than opium in large and repeated doses, by the method of Huetius, as taught to me by Dr. Physick. There is here a strong analogy to delirium tremens.

I am informed that Dr. Wm. H. Klapp employs emetics very successfully in the Moyamensing prison. I understand his cases include a large portion of instances of recent drunkenness. For this I have used emetics with much advantage, but not for delirium with phantoms. As a part of what is in this country called the antiphlogistic treatment, purgatives have been recommended in delirium tremens. I think it best to postpone their employment until after the *opium* has relieved the delirium. In Huetius's treatment for insanity, purgatives are directed to be given only every seven days. In delirium with phantoms, it will, I think, be practically found that purgatives, administered before the patient has had rest, do more injury by interfering with that requisite repose, than compensates for the benefit they afford.

### DOMESTIC SUMMARY.

In consequence of communications between members of the American Philosophical Society, in Philadelphia, and gentlemen in Boston, a meeting was held in the latter place, of gentlemen belonging to Boston, Salem, and the Uni-

* This refers to Professors Joseph Frank and Speranza.

